# Risk factors of sexually transmitted infections among female sex workers in Republic of Korea

**DOI:** 10.1186/s40249-019-0516-x

**Published:** 2019-01-22

**Authors:** Minsoo Jung

**Affiliations:** 1 Department of Health Science, College of Natural Science, Dongduk Women’s University, 23-1 Wolgok-dong, Seongbuk-gu, Seoul, 136-714 Republic of Korea; 2 Center for Community-Based Research, Dana-Farber Cancer Institute, Boston, USA

**Keywords:** Female sex worker, Sexual risk behaviour, Sexual transmitted infection, Republic of Korea

## Abstract

**Background:**

Female sex workers (FSW) who live in urban areas in Republic of Korea have a particularly high risk of sexually transmitted diseases (STD). We investigated the prevalence of s STDs in FSWs in order to determine the factors associated with sexually transmitted infections in Korea.

**Methods:**

Study data were collected from 832 FSWs through a 2014 survey on the prevalence of STDs in high risk populations in Republic of Korea. We assessed the associations between sexually transmitted infections and demographic and risk behaviour variables through logistic regression analysis.

**Results:**

The risk probability of sexually transmitted infection was higher for those who drank alcohol often as well as those who had their first sexual experience at an early age. However, the effects of these factors were attenuated by working conditions. The risk probability of sexually transmitted infections was higher for those who engaged in sex with several customers per day as well as for those who did not practice regular condom use.

**Conclusions:**

The risk factors for sexually transmitted infections among FSWs in Republic of Korea are related to and affected by working conditions. Thus, multifaceted health interventions to protect FSWs and their sexual health are deemed necessary.

**Electronic supplementary material:**

The online version of this article (10.1186/s40249-019-0516-x) contains supplementary material, which is available to authorized users.

## Multilingual abstracts

Please see Additional file 1 for translations of the abstract into the five official working languages of the United Nations.

## Background

Sexually transmitted infections (STIs) are passed from person to person through intimate sexual contact [1]. Untreated STIs may result in acute diseases, infertility, disabilities, and even death [2]. Moreover, STIs are difficult to prevent, due to difficulties accessing the groups that are particularly vulnerable to sexually transmitted diseases (STDs). These groups, so-called “unseen communities,” are particularly prevalent in Asia, where sexual activities can be deemed distasteful and where the social stigma attached to sexual activities may be particularly strong [3, 4]. Nevertheless, public health intervention is necessary because there are groups of people who have sexual relations with many partners and do not regularly use condoms [5, 6]. In developed countries in Asia, the prevalence of STIs tends to be higher in urban areas as well as among unmarried individuals and young adults, including female sex workers (FSWs) [7].

During the rapid industrialization of Republic of Korea starting in the 1960s, it condoned the expansion of the sex industry under its Confucian patriarchal system [7–9]; consequently, large cities have seen the formation of red-light districts (RLDs). In 2006, an estimated 3.5% of the female adult urban population was engaged in commercial sexual transactions [10]. FSWs who live in urban areas have a particularly high risk of STD infections, including gonorrhea (*Neisseria gonorrhea*), syphilis (*Treponema pallidum*), and chlamydia (*Chlamydia trachomatis*) [11]. The poor working conditions experienced by FSWs can harm their sexual health and lead to psychological problems such as anxiety disorders, personality disorders, and depression [12–14].

Since the implementation of the Act on the Prevention of Acts of Arranging Sexual Traffic in January 2005, the Republic of Korea government has increased its suppression of prostitution in brothels. As a result, many such business establishments have closed. Despite this, FSWs are still active in Republic of Korea. The present study investigated the prevalence of syphilis, chlamydia, and gonorrhea in these workers and determined the factors associated with such STD infections in Korean FSWs.

## Methods

### Ethics statement

Ethics and governance approvals were awarded by the Institutional Review Board, Korea Centers for Disease Control and Prevention (January 22, 2014; 20 140 122). In order to protect the vulnerable research subjects, actual site interviews and investigations were performed after receiving written informed consent from each of the participants. During the investigation process, absolutely no identifiable information was collected.

### Study design

We conducted cross-sectional study surveys between May 2 and November 27, 2014 through a collaboration with the Korea Federation for HIV/AIDS Prevention (KAIDS). To begin, representatives from KAIDS contacted business owners of 14 representative sex work venues identified via a 2008 nationwide economic survey of the sex industry. Ten of the 14 venue owners responded positively to the request to participate in our study. Of these 10 venues, due to sampling convenience inefficiencies, we excluded four small venues. Sample size determination was based on the assumption that the target FSW population was 4500, as reported in 2008 by the Korean Ministry of Gender Equality. Based on that estimate, the required sample size was 800 FSWs, assuming a chlamydia prevalence of 30% and a precision of 2.5% [7, 15].

### Data collection

The study data were collected through a 2014 survey of 832 respondents entitled Prevalence of sexually transmitted diseases in high risk populations of Republic of Korea, 2014 (*n* = 832). Individual surveys were conducted through 10- to 20- minute interviews administered and performed by a team consisting of a physician, two clinical pathologists, an individual with a master’s degree in public health, and a KAIDS director. The trained interviewers encouraged FSWs to answer the survey’s questions on sexual behaviours, and laboratory technicians obtained urine and blood samples from the participating FSWs.

Biological samples were obtained and tested as follows: serum TP-PA test for *T. pallidum,* and urine PCR for the presence of *C. trachomatis* or *N. gonorrhea*. FSWs who provided written informed consent to participate in the study (878) were included, while those who refused to sign (46) were excluded; the response rate for the survey was 94.8%. HIV screening, Western blot, for female sex workers (FSWs) was performed, but the prevalence rate was so low that it was excluded from the statistical analysis (1 out of 832). After a manual review of the completed questionnaires, the data were input via a double entry system. Urine samples were analysed at a central laboratory with a commercial PCR kit (BDProbTecET, BD Diagnostic Systems, Becton Dickinson, United Kindom). Pathological examination of the clinical specimens was conducted at a community health centre near each survey district, in Republic of Korea. All surveys and examinations were carried out with the approval of the Korea Centers for Disease Control and Prevention.

### Measures

The measurements used for each of the study variables were based on those described in the abridged version of the Behavioral Surveillance Surveys, an internationally validated survey approach [16].

#### Dependent variable

Regarding STI status, all participants currently infected or previously diagnosed with active syphilis, chlamydia, or gonorrhea were included. Current infections were identified through clinical examinations, while past infections were identified by physicians through survey questions asked during each participant’s examination. The STI status question responses were coded as ‘1’ if the pertinent results were positive and ‘0’ if the pertinent results were negative.

#### Independent variables

The age at sexual initiation was determined by asking whether participants had their first sexual intercourse experience prior to or after the age of 18. Sexual working conditions were assessed by determining the number of customers and the frequency of customer condom use. The number of customers was determined by asking the closed-ended question: How many customers did you have per day on average? The frequency of condom use was assessed by asking: how often did you use condoms with your customers during the last month? The responses were categorized as always, usually, often, and sometimes. Physical health was assessed by determining the participant’s self-rated health (SRH) status. SRH status is a useful predictor of health status in the fields of public health and epidemiology. Although this relatively simple health rating approach cannot capture the full dimensions of clinical health status, it has been reported to reliably predict survival in vulnerable populations, even after allowing for the effects of known health risk factors [17, 18]. In order to assess SRH, we asked: in general, would you say your health is (1) good, (2) average or (3) poor? Participant depression was also assessed by asking: Have you ever experienced severe depression for 2 weeks or more after becoming a FSW? The responses were yes or no. In order to reduce the potential for social desirability bias, we used validity checks to measure internal consistency. The survey questions regarding participation in risky health behaviors were based on the World Health Organization’s instrument for determining degrees of tobacco smoking and alcohol consumption [19].

#### Covariates

Covariates were selected based on theoretically and empirically defined relationships with STD infections [7]. Educational level attainment was assessed based on responses to: What is the highest level of school you attended?; the responses categories included middle school, high school to associate, and college or higher education level. Republic of Korea has compulsory education until middle school, so educational levels below middle school were excluded. Participant ages (years) were categorized as follows: 19–24, 25–29, 30–34, 35–39, and 40 and older.

### Statistical analysis

Participant responses with missing values for key analysis variables were excluded using a pairwise method. After these exclusions, the study sample consisted of 774 FSWs with urine and blood sample results. Data from those participants were used in the study’s multivariate analyses. We estimated the descriptive statistics of the survey participants’ prevalences of active syphilis*, Chlamydia trachomatis*, and *Neisseria gonorrhea*. The Pearson chi-square test was used to examine the association between STI prevalence and individual factors. We assessed the association between STI prevalence and demographic and risk behaviour variables via binary logistic regression analysis. We entered all variables that have been identified by other studies as significant factors into the model [7, 8]. We calculated the odds ratios (*OR*s) and 95% confidence intervals (*CI*s) for the demographic and sexual risk behaviour variables. Statistical analyses were performed using SPSS version 21.0 (IBM SPSS Institute, Chicago, IL, US).

## Results

### Participant characteristics

Table 1 shows that 65.1% of the respondents were in the high school graduate to associate category of education, 82.9% of the respondents were current smokers, and 19.3% drank alcohol three times or more per week. In addition, 9.1% of the participants had a poor SRH status and 28.1% reported experiencing depression. The majority of FSWs had their first sexual experience at an early age: 53.7% experienced their first sexual experience as minors (under 18 years old). In terms of customers, 29.4% had four to five sex partners per day, and only 22.6% used condoms with every sex partner. Of the 832 participants, 38.2% reported they were infected with STDs, and 61.1% reported they had never been infected by STIs. The current prevalences of syphilis, gonorrhea, and chlamydia were determined from the 774 FSWs who had submitted both urine and blood samples. Of those FSWs, 2.6% were syphilis-positive (95% *CI*: 1.47–3.72%), 1.0% were gonorrhea-positive (95% *CI*: 0.28–1.71%), and 10.9% were chlamydia-positive (95% *CI*: 8.70–13.09%) (Table 2).

**Table 1:** General sample characteristics (*n* = 832)

	*n*	%		*n*	%
Age			Sexual initiation		
19–24	40	4.8	Before 18	447	53.7
25–29	163	19.6	After 19	309	37.1
30–34	283	34.0	Missing	76	9.1
35–39	212	25.5	Total	832	100.0
40 or older	128	15.4	Average number of customer (per day)		
Missing	6	0.7	3 or less	258	31.0
Total	832	100.0	4–5	245	29.4
Education			6–9	81	9.7
Middle school	89	10.7	10 or more	43	5.2
High school to associate	542	65.1	Missing	205	24.6
College or higher	122	14.7	Total	832	100.0
Missing	79	9.5	Condom use		
Total	832	100.0	Always	188	22.6
Smoking			Usually	372	44.7
Nonsmoker	100	12.0	Often	183	22.0
Smoker	690	82.9	Sometimes	40	4.8
Missing	42	5.0	Missing	49	5.9
Total	832	100.0	Total	832	100.0
Drinking alcohol			STIs infection experience		
Almost everyday	71	8.5	Negative	508	61.1
3–4 times/wk	90	10.8	Positive	318	38.2
1–2 times/wk	218	26.2	Missing	6	0.7
2–4 times/mo or less	393	47.2	Total	832	100.0
Missing	60	7.2	Depression		
Total	832	100.0	Have not	558	67.1
Self-rated health (SRH)			Have	234	28.1
Good	366	44.0	Missing	40	4.8
Average	347	41.7	Total	832	100.0
Poor	76	9.1			
Missing	43	5.2			
Total	832	100.0			

*STI* Sexually transmitted infection

**Table 2:** Prevalence of sexually transmitted infections by recruitment regions among female sex workers in Republic of Korea, 2014

	Syphilis		Gonorrhea		Chlamydia	
	Positive (*n*)	Percentage (%)	Positive (*n*)	Percentage (%)	Positive (*n*)	Percentage (%)
Seoul metropolitan city (Capital)						
A (*n* = 53)	3	(5.7)	1	(1.9)	4	(7.5)
B (*n* = 128)	10	(7.8)	0	(0.0)	16	(12.5)
C (*n* = 60)	5	(8.3)	0	(0.0)	6	(10.0)
D (*n* = 99)	0	(0.0)	1	(0.1)	11	(11.1)
Gyeonggi province						
E (*n* = 68)	0	(0.0)	1	(1.5)	6	(8.8)
F (*n* = 67)	1	(1.5)	0	(0.0)	8	(11.9)
Gangwon province						
G (*n* = 18)	0	(0.0)	1	(5.6)	2	(11.1)
Gyeonsang province						
H (*n* = 132)	1	(0.8)	1	(0.8)	11	(8.3)
I (*n* = 52)	0	(0.0)	3	(5.8)	4	(7.7)
Jeolla province						
J (*n* = 97)	0	(0.0)	0	(0.0)	16	(16.5)
Total (*N* = 774)	20	(2.6)	8	(1.0)	84	(10.9)

Note: only the sample who participated in both urine and blood tests were considered as a denominator

### Univariate analysis of STI prevalence by demographic characteristics

The differences in FSWs’ characteristics in terms of STD infection experience were analysed using the Pearson’s *χ*^2^ test (Table 3). Significant differences in STI prevalence were detected between the proportions of FSWs within the relevant categories for alcohol consumption (*P* < 0.05), experience with depression after beginning sex work (*P* < 0.01), SRH status (*P* < 0.01), age of first sexual intercourse (*P* < 0.001), daily number of sex customers (*P* < 0.05), and frequency of condom use (*P* < 0.001).

**Table 3:** Univariate analyses of STI infection by socio-demographics and sexual working conditions of FSWs in Republic of Korea, 2014

		STDs Infection				*χ*^2^ *P*-value			STDs Infection				*χ*^2^ *P*-value
		Negative (*n*, %)		Positive (*n*, %)					Negative (*n*, %)		Positive (*n*, %)		
Age	19–24	23	57.5%	17	42.5%	n.s.	Depression	Have	123	52.6%	111	47.4%	*P* < 0.01
	25–29	97	59.5%	66	40.5%			Have not	353	63.3%	205	36.7%	
	30–34	172	61.2%	109	38.8%			Total	476	60.1%	316	39.9%	
	35–39	133	63.6%	76	36.4		Self-rated health	Bad	240	65.6%	126	34.4%	*P* < 0.01
	40 or more	78	61.4%	49	38.6%			Average	191	55.0%	156	45.0%	
	Total	503	61.3%	317	38.7%			Good	42	55.3%	34	44.7%	
Education	Middle school	53	59.6%	36	40.4%	n.s.		Total	473	59.9%	316	40.1%	
	High school to associate	321	59.2%	221	40.8%		Sexual initiation	Before 18	166	53.7%	143	46.3%	*P* < 0.001
	College or higher	77	63.1%	45	36.9%			After 19	292	65.3%	155	34.7%	
	Total	451	59.9%	302	40.1%			Total	458	60.6%	298	39.4%	
Smoking	Smoker	63	63.0%	37	37.0%	n.s.	Number of sex customers (per a day)	3 or less	171	66.3%	87	33.7%	*P* < 0.05
	Non-smoker	411	59.6%	279	40.4%			4–5	143	58.4%	102	41.6%	
	Total	474	60.0%	316	40.0%			6–9	41	50.6%	40	49.4%	
Drinking alcohol	Almost everyday	35	49.3%	36	50.7%	*P* < 0.05		10 or more	21	48.8%	22	51.2%	
	3–4 times/week	46	51.1%	44	48.9%			Total	376	60.0%	251	40.0%	
	1–2 times/week	132	60.6%	86	39.4%		Condom use	Always	147	78.2%	41	21.8%	*P* < 0.001
	2–4 times/more or less	249	63.4%	144	36.6%			Usually	215	57.8%	157	42.2%	
	Total	462	59.8%	310	40.2%			Often	95	51.9%	88	48.1%	
								Sometimes	14	35.0%	26	65.0%	
								Total	471	60.2%	312	39.8%	

*STDs* Sexually transmitted diseases, *STI* Sexually transmitted infection, *FSWs* Female sex workers

*n.s* not significant

### Multivariate analyses of STI prevalence by socio-demographic and working conditions

The results of our binary logistic regression analyses indicated that, after adjusting for socio-demographic factors such as age, educational level attainment, and recruitment region (Table 4), the risk probability for an STD infection was higher among participants who drank alcohol more often than among those who drank less often (Model II; a*OR* = 1.21; 95% *CI*: 1.04–1.41), and the STD infection risk was higher among those who had their first sexual experience under the age of 18 than among those who had their first sexual experience when they were older (Model IV; a*OR* = 1.41; 95% *CI*: 1.01–1.98). The association between drinking alcohol and STIs prevalence remained significant when SRH status was included in the model (Model III), as well as when age at sexual initiation was included in the model (Model IV). The effect of alcohol consumption on STI prevalence was changed insignificantly when working conditions were included in the model (Model V). However, the risk probability of an STD infection was higher for those who had more sex customers per day than in those who had fewer daily customers (a*OR* = 1.27; 95% *CI*: 1.04–1.56). Similarly, the STD infection risk was higher for those who did not practice regular condom use than for those who regularly used condoms (a*OR* = 1.67; 95% *CI*: 1.33–2.10).

**Table 4:** Multivariate analyses of STI infection by socio-demographics, health and sexual risky behaviours of FSWs in Republic of Korea, 2014

	Model I			Model II			Model III			Model IV			Model V		
	a*OR*	95% *CI*		a*OR*	95% *CI*		a*OR*	95% *CI*		a*OR*	95% *CI*		a*OR*	95% *CI*	
		Lower	Upper		Lower	Upper		Lower	Upper		Lower	Upper		Lower	Upper
Socio-demographic Characteristics															
Age	0.94	0.83	1.08	0.95	0.83	1.09	0.96	0.83	1.10	1.02	0.87	1.18	1.00	0.84	1.19
Education	0.89	0.67	1.18	0.90	0.68	1.20	0.92	0.69	1.23	0.95	0.71	1.28	0.85	0.61	1.18
Health Behavior															
Smoking				1.05	0.67	1.65	1.00	0.63	1.57	0.96	0.60	1.53	0.99	0.58	1.69
Drinking alcohol				1.21*	1.04	1.41	1.19*	1.02	1.39	1.21*	1.03	1.42	1.10	0.92	1.31
Health Status															
Self-rated health							1.18	0.93	1.51	1.13	0.88	1.46	0.95	0.71	1.27
Depression							1.33	0.94	1.88	1.31	0.92	1.88	1.32	0.88	1.97
Sexual Initiation															
Before the age of 18										1.41*	1.01	1.98	1.17	0.80	1.72
Working Conditions															
Number of sex customers													1.27*	1.04	1.56
Unsafe sex													1.67**	1.33	2.10

a*OR* Adjusted odds ratio, *CI* Confidence interval, *FSWs* Female sex workers, *STI* Sexually transmitted infection

All models are additionally adjusted for the baseline factor of recruitment locations

*P < 0.05; **P < 0.001

## Discussion

Republic of Korea began a crackdown on the country’s sex industry and enacted a new anti-prostitution law in January 2005 that was aimed at brothel owners, prostitutes, and their clients. The objective of this study was to investigate STIs prevalence and its associations with sexual-health related factors among FSWs in Republic of Korea. The results indicate that the probability of having an STI was significantly associated with whether the FSWs exercised safe sex practices (i.e., used condoms) and had a limited number of daily sex partners. In addition, STI status was related to risky health behaviour (i.e., alcohol consumption frequency) and age.

The present study has three implications. First, the unstable legal positions of FSWs working outside of business venues can make it difficult for them to practice safe sex. This is exhibited by the fact that since the anti-prostitution legislation was enacted, the use of condoms by FSWs living in RLDs has decreased [7]. Prior to the legislation, there were tacit norms where brothel owners and FSWs made it compulsory for sex clients to use condoms; thus, the sexual health of FSWs was protected to some extent. However, since the passage of the act, sex clients often coerce FSWs into engaging in illegal acts (i.e., prostitution) and may persuade them to provide services associated with high risks of infection, such as oral sex and/or sex without a condom [20]. Our results show that the risk probability of having an STD infection was 1.67 times higher for those who did not practice regular condom use than for those that regularly used condoms. Thus, many present-day FSWs in Republic of Korea are engaging in risky behaviours under poor working conditions.

Second, working conditions rather than individual characteristics may be the more fundamental causes of STIs among FSWs. Variables such as alcohol consumption and the age of first sexual experience were statistically significant in Model IV but had no effect in Model V. In contrast, when variables related to FSW working conditions were analysed, the number of sex clients per day was a significant determinant of STD infection status, and it was also a more important factor than the other individual characteristics of FSWs, such as alcohol consumption and age of first sexual experience. The sex industry in Republic of Korea, like that in other developed Asian countries, has been undergoing reorientation from direct to indirect prostitution [21]. This change was anticipated with the enactment of Republic of Korea’s anti-prostitution law, which has resulted in a so-called ‘balloon effect’ that has caused changes in the structure and visibility of prostitution, including the migration of FSWs to neighbouring countries and the participation of FSWs in indirect sex work via informal regional linkages within the sex industry. Therefore, even though the anti-prostitution act may have reduced the number of prostitutes, the act may have not contributed to any reduction in STD infections among FSWs. Furthermore, since the enactment of the law, sex-traffic related health budgets have been reduced by 15–20%. In particular, the entire budget for medical check-ups for STD infections among FSWs in brothels has been retrenched [22]. Thus, present-day FSWs in Republic of Korea may be penalized not only through the enforcement of the anti-prostitution act, but also by a health budget that is inadequate to monitor the incidence rates of STD infections.

Third, the results of the present study indicate that there is a need to implement community-based health interventions for STD infections among subpopulations that engage in risky or unprotected sex [23]. Many of the groups among the general population with high STD infection rates have concurrent sexual partnerships, consume alcohol frequently, inconsistently use condoms, and began their sexual experiences at an early age [24]; such characteristics are similar to those of FSWs in Republic of Korea [7, 8]. Regardless, there have been few reports on the risk factors for STD infections among FSWs in countries where prostitution is illegal. Moreover, the balloon effect of Republic of Korea’s anti-prostitution law may have resulted in the spread of prostitution into residential areas. Our results suggest that the high prevalence of STIs among Republic of Korean FSWs may be associated with engagement in risky sex behaviours. Thus, there is a need to use community-based approaches to compare STI rates in FSWs in countries in which prostitution is legal with those where prostitution is illegal. Several limitations of the present study should be noted. First, the limited availability of information on the size and distribution of the FSW population in Republic of Korea made it impossible for us to conduct probabilistic sampling, which may have decreased the external validity of our results. However, the large sample size of this study from a so-called underground population, with a sample obtained by covering most major FSW sites in Republic of Korea and with the approval of Korean government, is a strength of this study. Second, the gatekeeper characteristics of the different RLDs and sex-traffic establishments surveyed in our study were not assessed [25]. Gatekeepers, who are most often the brothel owners, can implement differing tacit norms for condom use and the number of daily clients, and variations in those norms may have affected our results. Third, we did not cover all types of FSWs, as our sample was limited to full-time FSWs. Analysing the differences between full-time and part-time FSWs may have produced different results [26].

## Conclusions

Since the passage of the Korean anti-prostitution act, FSWs who remain active continue to participate in illegal activities and expose themselves to risky working conditions. The results of the present study show that the risk factors for STD infections among FSWs in Republic of Korea are mainly related to working conditions. This is in part due to FSWs being coerced to provide services demanded by clients, with some FSWs practicing unprotected sex [11]. The present study reveals that the working conditions of FSWs, who are even more vulnerable today because of the provisions of the anti-prostitution act and the associated reductions to sex-traffic-related health budgets, are important factors associated with the incidences of STD infections. Thus, multifaceted community-based interventions to protect FSWs and their sexual health are necessary. In terms of public health, making the entire sex industry illegal may not be the best method for reducing STD infections rates among FSWs.

## Additional file

Additional file 1: Multilingual abstracts in the five official working languages of the United Nations. (PDF 390 kb)

## Abbreviations

*CI*s: Confidence intervals
FSWs: Female sex workers
KAIDS: Korea Federation for HIV/AIDS Prevention
*OR*s: Odds ratios
RLDs: Red-light districts
SRH: Self-rated health
STD: Sexually transmitted disease
STI: Sexually transmitted infection
