# Supplementary material for: Risk factors of sexually transmitted infections among female sex workers in Republic of Korea
Source: Infect Dis Poverty. 2019 Jan 22;8:6. doi: 10.1186/s40249-019-0516-x (PMC6341713; doi:10.1186/s40249-019-0516-x)
Supplement: Supplementary file 1 — Multilingual abstracts in the five official working languages of the United Nations. (PDF 390 kb) [file 40249_2019_516_MOESM1_ESM.pdf]

Translation of the abstract into the five official working languages of the United Nations

مخاطر الإصابة بالأمراض المعدية المنقولة جنسيًا لدى النساء المُشتغلات بالجنس في كوريا الجنوبية

مينسو يانج

الملخص

خلقية الموضوع: تزيد مخاطر الإصابة بالعدوى المنقولة جنسيًا (STD) لدى النساء المُشتغلات بالجنس (FSW) اللاتي يقطن في المناطق الحضارية في كوريا الجنوبية بصفة خاصة. أجرينا دراسة لقياس مدى انتشار الإصابة بالأمراض المعدية المنقولة جنسيًا لدى النساء المُشتغلات بالجنس من أجل الوقوف على العوامل ذات الصلة بانتقال العدوى في هذا السياق في كوريا.

المنهجية: شملت هذه الدراسة بيانات 832 سيدة من المُشتغلات بالجنس جُمعت خلال استطلاع تم إجراءه عام 2014 حول مدى انتشار الأمراض المعدية المنقولة جنسيًا ضمن التجمعات الأكثر عرضة لذلك في كوريا. حددنا الرابط بين الأمراض المعدية المنقولة الجنسيًا وبين المتغيرات الديموغرافية والسلوكيات شديدة الخطورة باتباع أسلوب تحليل الارتباط المنطقي.

النتائج: أظهرت النتائج أن احتمالات الإصابة بالعدوى المنقولة جنسيًا أعلى بمعدل متماثل لدى الأشخاص المسرفين في تناول المشروبات الكحولية ولدى من بدأوا ممارسة الجنس في سن صغيرة. بالرغم من ذلك لم تُقلص تأثيرات تلك العوامل ضمن ظروف العمل إلا على نحوٍ يسير. أظهرت أيضًا أن احتمالات الإصابة بالعدوى المنقولة جنسيًا أعلى بمعدل متماثل لدى من مارسن الجنس مع عدة أشخاص في اليوم الواحد ولدى من لم يعتمدوا استعمال وسائل الحماية الاعتيادية.

الاستنتاجات: تتعلق مخاطر الإصابة بالعدوى المنقولة جنسيًا لدى النساء المُشتغلات بالجنس في كوريا الجنوبية بظروف العمل وتتأثر بها. بناءًا على ذلك، بات إعداد تدخلات صحية متعددة الأوجه لوقاية النساء المُشتغلات بالجنس وحماية سلامتهن الجنسية ضرورة.

Translated from English version into Arabic by Suzan Alkhodair and Aliaa, through

TRANSLATORS
WITHOUT BORDERS

韩国女性性工作者感染性传播疾病的风险因素

Minsoo Jung

摘要

引言：韩国城市地区的女性性工作者（FSW）感染性传播疾病（STD）的风险非常高。为明确韩国 STD 的传播相关因素，我们在女性性工作者中调查了 STD 的流行情况。

方法：2014 年在韩国流行区高危人群中调查了 832 名女性性工作者。通过 logistic 回归分析评估了 STD 与人口统计学及高危行为变量之间的相关性。

结果：酗酒和早年发生首次性行为的人群感染 STD 的风险更高。而且这些因素受工作环境的变化影响。性工作频繁以及不经常使用避孕套的人员感染 STD 的风险更高。

结论：韩国女性性工作者中感染 STD 的风险与工作条件相关并受其影响。因此，有必要采取多重健康干预措施来保护女性性工作者及其生理健康。

Translated from English version into Chinese by Peng Song, edited by Jin Chen

CHINESE CENTER FOR DISEASE CONTROL AND PREVENTION
NATIONAL INSTITUTE OF PARASITIC DISEASES

Facteurs de risque des infections sexuellement transmissibles chez les travailleuses sexuelles en Corée du Sud

Minsoon Jung

Résumé

Contexte : Le risque d'infection par des maladies sexuellement transmissibles (MST) est particulièrement élevé chez les travailleuses sexuelles qui vivent dans des zones urbaines de la Corée du Sud. Nous avons étudié la prévalence des MST chez les travailleuses sexuelles afin de déterminer les facteurs associés aux infections sexuellement transmissibles en Corée.
Méthodes : Les données de l'étude ont été recueillies auprès de 832 travailleuses sexuelles lors d'une enquête menée en 2014 portant sur la prévalence des MST dans les populations à haut risque en Corée. Nous avons évalué les associations entre les infections sexuellement transmissibles et les variables démographiques, ainsi que les comportements à risque via une analyse de régression logistique.
Résultats: La probabilité du risque d'infection sexuellement transmissible était supérieure pour celles qui buvaient de l'alcool souvent, ainsi que celles qui avaient vécu leur première expérience sexuelle à un âge précoce. Cependant, les effets de ces facteurs étaient atténués par les conditions de travail de façon non significative. La probabilité du risque d'infections sexuellement transmissibles était supérieure pour celles qui avaient des relations sexuelles avec plusieurs clients par jour ainsi que celles qui ne pratiquaient pas l'utilisation régulière de préservatifs.
Conclusions : Les facteurs de risque pour les infections sexuellement transmissibles chez les travailleuses sexuelles en Corée du Sud sont liés à leurs conditions de travail et influencés par ces dernières. Par conséquent, nous estimons que des interventions sanitaires aux multiples facettes sont nécessaires pour protéger les travailleuses sexuelles et leur santé sexuelle.

Translated from English version into French by anne-elise and Suzanne Assenat, through

TRANSLATORS
WITHOUT BORDERS

Факторы риска инфекций, передаваемые половым путем у женщин, работниц секс-бизнеса в Южной Корее.

Минсо Чун

Краткое описание:

Предпосылка: У секс-работниц (ЖСР), проживающих в городских районах Южной Кореи, крайне высокий риск заболеваний инфекциями, передающимися половым путем (ЗППП). Мы изучили число случаев ЗППП у ЖСР, с целью определения факторов, влияющих на данное явление в Корее.

Методы: Мы получили данные о распространении ЗППП среди групп повышенного риска в Корее, благодаря проведённому опросу 832 ЖСР в 2014 году. А также на основе метода логистической регрессии проанализировали влияние инфекций, передаваемых половым путем на демографические переменные и переменные, связанные с риском поведения.
Результаты: Вероятность распространения инфекций, передаваемых половым путем, была выше у тех, кто часто употреблял алкоголь, а также у тех, кто имел первый сексуальный опыт в раннем возрасте. Однако как оказалось, рабочие условия имеют решающее значение вдобавок к указанным факторам. Вероятность распространения инфекций, передаваемых половым путем, была выше у тех, кто занимался сексом с несколькими клиентами в день, а также для тех, кто регулярно не пользовался презервативами.
Выводы: Факторы риска инфекций, передаваемые половым путем у работниц секс-бизнеса в Южной Корее, зависят от условий труда. Таким образом, необходимо принять комплексные медико-санитарные меры для защиты ЖСР и их сексуального здоровья.

Translated from English version into Russian by Irina Stenina and Liudmila Tomanek, through

TRANSLATORS WITHOUT BORDERS

Factores de riesgo de infecciones de transmisión sexual en las trabajadoras sexuales de Corea del Sur

Minsoo Jung

Resumen
Antecedentes: Las trabajadoras sexuales (FSW, por sus siglas en inglés) que viven en las zonas urbanas en Corea del Sur tienen un riesgo particularmente alto de enfermedades de transmisión sexual (ETS). Se investigó la prevalencia de ETS en las FSWs para determinar los factores asociados con las infecciones de transmisión sexual en Corea.
Métodos: Se recolectaron datos del estudio de 832 FSWs a través de una encuesta de 2014 sobre la prevalencia de ETS en poblaciones de alto riesgo en Corea. Evaluamos las relaciones entre las infecciones de transmisión sexual y las variables demográficas y de comportamiento de riesgo a través del análisis de regresión logística.
Resultados: La probabilidad de riesgo de una infección de transmisión sexual fue mayor para quienes bebían alcohol a menudo, así como para quienes tuvieron su primera experiencia sexual a una edad temprana. Sin embargo, los efectos de estos factores fueron atenuados de manera insignificante por las condiciones de trabajo. La probabilidad de riesgo de infecciones de transmisión sexual fue mayor para quienes tuvieron relaciones sexuales con varios clientes por día, así como para quienes no emplearon preservativos regularmente.
Conclusiones: Los factores de riesgo para las infecciones de transmisión sexual entre las FSW en Corea del Sur están relacionados y se ven afectados por las condiciones laborales. De este modo, se consideran necesarias las intervenciones de salud multifacéticas para proteger a las FSW y su salud sexual.

Translated from English version into Spanish by Aileen Nieto andJaime Fernández Gianzo, through

TRANSLATORS
WITHOUT BORDERS
